# Opioid prescribing patterns for non-malignant chronic pain for rural versus non-rural US adults: a population-based study using 2010 NAMCS data

**DOI:** 10.1186/s12913-014-0563-8

**Published:** 2014-11-19

**Authors:** Jacob P Prunuske, Catherine A St. Hill, Keri D Hager, Andrine M Lemieux, Michael T Swanoski, Grant W Anderson, M Nawal Lutfiyya

**Affiliations:** Department of Family Medicine and Community Health, School of Medicine, University of Minnesota, Duluth, MN USA; Department of Experimental and Clinical Pharmacology, College of Pharmacy, University of Minnesota, 308 Harvard St. SE, Minneapolis, MN USA; Department of Pharmacy Practice and Pharmaceutical Sciences, College of Pharmacy, University of Minnesota, 1033 Kirby Drive, Duluth, MN USA; Behavioral Medicine Laboratory, School of Medicine, University of Minnesota, 1033 Kirby Drive, Duluth, MN USA; National Center for Interprofessional Education and Practice, University of Minnesota, R685 Children’s Rehab Center, 426 Church Street SE, Minneapolis, MN 55455 USA

**Keywords:** NAMCS data, Rural health, Health care disparities, Opioids, Non-malignant chronic pain, NMCP

## Abstract

**Background:**

Non-malignant chronic pain (NMCP) is one of the most common reasons for primary care visits. Pain management health care disparities have been documented in relation to patient gender, race, and socioeconomic status. Although not studied in relation to chronic pain management, studies have found that living in a rural community in the US is associated with health care disparities. Rurality as a social determinant of health may influence opioid prescribing. We examined rural and non-rural differences in opioid prescribing patterns for NMCP management, hypothesizing that distinct from education, income, racial or gender differences, rural residency is a significant and independent factor in opioid prescribing patterns.

**Methods:**

2010 National Ambulatory Medical Care Survey (NAMCS) data were examined using bivariate and multivariate techniques. NAMCS data were collected using a multi-stage sampling strategy. For the multivariate analysis performed the SPSS complex samples algorithm for logistic regression was used.

**Results:**

In 2010 an estimated 9,325,603 US adults (weighted from a sample of 2745) seen in primary care clinics had a diagnosis of NMCP; 36.4% were prescribed an opioid. For US adults with a NMCP diagnosis bivariate analysis revealed rural residents had higher odds of having an opioid prescription than similar non-rural adults (OR = 1.515, 95% CI 1.513-1.518). Complex samples logistic regression analysis confirmed the importance of rurality and yielded that US adults with NMCP who were prescribed an opioid had higher odds of: being non-Caucasian (AOR =2.459, 95% CI 1.194-5.066), and living in a rural area (AOR =2.935, 95% CI 1.416-6.083).

**Conclusions:**

Our results clearly indicated that rurality is an important factor in opioid prescribing patterns that cannot be ignored and bears further investigation. Further research on the growing concern about the over-prescribing of opioids in the US should now include rurality as a variable in data generation and analysis. Future research should also attempt to document the ecological, sociological and political factors impacting opioid prescribing and care in rural communities. Prescribers and health care policy makers need to critically evaluate the implications of our findings and their relationship to patient needs, best practices in a rural setting, and the overall consequences of increased opioid prescribing on rural communities.

## Background

Chronic pain, commonly defined as the experience of episodic or continuous debilitating pain over time (weeks to months or longer), is a significant problem in the United States [1–4]. It is a phenomenon with a complex etiology manifest with both physical and psychological components [4–6]. At present nearly 1 in 3 or 100 million adults in the US suffer from chronic pain [4,6]. Chronic pain in the US is estimated to cost approximately $560-$635 billion annually, an amount equal to about $2,000 per capita [6]. This cost estimate includes both direct and indirect costs that are approximately equal to one another.

Chronic pain adversely impacts communities, families, and individuals by significantly contributing to poor mental and physical health resulting in lost work productivity and disability [4–7]. In the US, non-malignant chronic pain (NMCP) is one of the most common reasons for primary care visits [1,3,8,9]. At times when patients visit a healthcare professional for NMCP, inadequate training and resources may prevent proper assessment and management [7,10,11]. Pain management healthcare disparities have been documented in relation to patient gender [12,13], race [3,11,14–16], and socioeconomic status [12,17]. Although not studied in relation to chronic pain management, studies have found that living in a rural community in the US is associated with healthcare disparities [18].

Opioids are commonly used in chronic pain management. Opioids are being overprescribed with possible negative consequences for individuals, families and communities. These consequences include: unintended death [7], overdose [7], diversion [7], and crime [7]. There is an upward trend for overprescribing opioids. Rurality as a social determinant of health may influence opioid prescribing [18]. Some research has been conducted on different types of chronic pain and opioid prescribing patterns including a geographic variable in the analysis. However, none of these studies undertook the task of answering a question about opioid prescribing and NMCP in rural versus non-rural adults using patient level health records [19–22]. This has created an epidemiological gap in our knowledge regarding opioid prescribing patterns for rural adults with NMCP.

Rasu, et al. [4] studied chronic pain management (medication and non-medication) in US ambulatory care settings. While they described the characteristics of their patient population, they did not examine the associations between patient characteristics and variations in prescribing patterns. They concluded that additional research should investigate patterns of NMCP management in various populations. Others [23,24] have done this, but with older data. One study included geographic location of physician practice site in their analyses using rural locale as the reference category in multivariate analyses and found no significant relationship to opioid prescribing [25]. In this paper we hypothesize that distinct from patient race, education, income or gender, rural residency is a significant and an independent risk factor for the greater probability of receiving an opioid prescription for NMCP.

## Methods

To answer the research question, 2010 National Ambulatory Medical Care Survey (NAMCS) data were examined using bivariate and multivariate techniques. NAMCS is designed to collect data on the utilization and provision of ambulatory care services nationwide. Data are collected from a national sample of ambulatory care visits. The survey employs a complex four-stage probability sampling design. A description of the sampling strategy is discussed elsewhere [25]. The 2010 NAMCS data were used for this study because they were the most recently available data. These data are weighted to be nationally representative of patient health records.

All analyses were performed on weighted data as is recommended by the Center for Disease Control and Prevention’s (CDC) National Center for Health Statistics (NCHS). The weighting, as calculated, uses the most recently available census data to provide a stratified representation of the nation’s patient population. Results report weighted data.

The survey uses a *Patient Record Form* as the survey instrument. The NAMCS patient record form is completed by ambulatory care staff for a systematic random sample of patient visits during a randomly assigned 1-week reporting period. Data are obtained on demographic characteristics of patients, expected source(s) of payment, patients’ complaints, diagnoses, diagnostic/screening services, procedures, medication therapy, disposition, types of providers seen, causes of injury, and certain characteristics of the facility, such as geographic region and metropolitan status.

Rurality, one of the key independent variables in this analysis, was derived using Metropolitan and Micropolitan Statistical Area (MSA) methodology. This is a definition used by federal level agencies for research purposes. MSA was recoded into the dichotomous categories of rural or non-rural. Rural residents were defined as people living either within an MSA that had no center city or outside an MSA. Non-rural residents included all respondents living in a center city of an MSA, outside the center city of an MSA but inside the county containing the center city, or inside a suburban county of an MSA.

The study population for this research was US adults with NMCP. NMCP is defined as pain lasting 3 months or more or as pain persisting beyond the time of expected healing. The three-digit ICD-9 code for NMCP is 338.2. The covariates or independent variables for this research were: geographic locale (rural/non-rural), patient sex (male/female), race/ethnicity (Caucasian/Non-Caucasian), age ranges (18-39/40-64/65 years and older), education attainment in a patient’s zip code (≤20% of adults with a university degree/> 20% of adults with a university degree), poverty level in patient’s zip code (<10%/≥ 10%) health insurance status (insured/noninsured), primary health care provider (HCP) seen (yes/no), depression diagnosis (yes/no), arthritis diagnosis (yes/no). Depression and arthritis diagnoses were included as variables because these may influence opioid prescribing [26,27]. All of the study covariates were recoded from their original configuration for analyses. Re-coding entailed either collapsing categories and/or removing unknown responses. Opioid prescription was the dependent variable for this study.

The study variable that entailed complicated re-coding was the dependent variable—opioid prescription. Using the CDC’s New Ambulatory Care Drug Database System for NAMCS Data, prescribed drugs were classified as opioid or other. Table 1 displays the opioid drug codes by generic drug name.

**Table 1:** **Opioid drug codes by generic drug name**

**Opioid drug codes from NAMCS drug database**	**Generic drug name**
25510, 5660, 8335	Propoxyphene
2387, 97062	Remifentanil
1187, 50040	Sufentanil
9286	Tapentadol
2333, 5081, 5091, 8246, 9582, 22303, 91047, 96109, 97181	Oxycodone
7117, 7223, 21575	Oxymorphone
23285, 30535, 30540	Pentazocine
8338	Phenerol
7420, 8475, 8490, 10115	Propoxyphene
91046, 92044, 92070, 98144, 99123	Morphine
21550, 60990	Nalbuphine
8606, 98067	Narcotic Analgesics
3064, 9969, 21860, 21870, 21875, 21880, 22720, 22845	Opium
22850	Opium-Sodium Bicarbonate
1288, 1314	Oxycodone
95085	Hydroxyzine-Meperidine
17340, 17362	Levorphanol
200, 8785, 18760, 96045	Meperidine
10130, 18985	Methadone
85, 2852, 3228, 8079, 10743, 19650, 19699, 26763, 41420, 60940, 70214	Morphine
91071	Dezocine
9574	Dihydrocodeine
2067, 3307, 7197, 9508, 29645, 60565, 92024, 94188	Fentanyl
14770, 92041, 92042	Homatropine Methyl Bromide-Hydrocodone
7582, 9435, 14955, 94184	Hydrocodone
9600, 9641, 15005	Hydromorphone
11225, 22415, 27315	Aspirin; Caffeine; Codeine; Phenacetin
11090, 18425, 24770, 25525	Aspirin; Caffeine; Phenacetin; Propoxyphene
8910	Atropine; Opium; Phenacetin; Salicylamide
5054, 60265, 95036	Buprenorphine
5103	Butalbital-Codeine
1021, 29285	Butorphanol
1028, 7180, 7185 7190	Codeine
25690	Codeine; Sanguinaria; Terpin Hydrate; White Pine Syrup; Wild Cherry Syrup
91012	Dezocine
10715	Acetaminophen; Aspirin; Caffeine; Dihydrocodeine
42245	Acetaminophen; Aspirin; Caffeine; Hydrocodone
40765	Acetaminophen; Butabarbital; Codeine
13152, 24143	Acetaminophen; Butalbital; Codeine
866, 96145	Alfentanil
21095	Alphaprodine
2730, 2735	Aluminum Hydroxide; Aspirin; Codeine; Magnesium Antacids
2740	Aluminum Hydroxide; Aspirin; Codeine; Magnesium Hydroxide
12560	Aspirin; Butalbital; Caffeine; Codeine; Phenacetin
45, 50, 55, 65, 1990, 2815, 2825, 11220	Aspirin; Caffeine; Codeine; Phenacetin
3520	Acetaminophen; Codeine; Salicylamide
6284	Acetaminophen; Ethanol; Glycerin; Hydrocodone; Parabens
250, 265, 270, 275, 280, 1758, 2340, 2345, 5151, 5640, 7080, 7165, 7618, 9538, 11265, 11268, 23665, 23670, 23675, 23680, 25635, 28215, 32910, 32915, 32920, 32925, 32930, 32935, 41245, 91010	Acetaminophen-Codeine
197	Acetaminophen-Dextropropoxyphene
10128, 40415	Acetaminophen-Dihydrocodeine
251, 1268, 1995, 2045, 2082, 2132, 2314, 3518, 6059, 7064, 8354, 10105, 14917, 34110, 40860, 60340, 61610, 89038, 89039, 92180, 93077, 93089, 96028, 96047, 98036, 98168	Acetaminophen-Hydrocodone
8790	Acetaminophen-Meperidine
283, 2348, 3394, 7251, 7252, 7632, 8248, 22305, 22306, 23385, 26958, 28272, 32945, 91048, 99114	Acetaminophen-Oxycodone
7701, 30513	Acetaminophen-Pentazocine
156, 6232, 8470, 25530, 25545, 28340, 34985, 61240, 89071, 89072, 93053, 93411	Acetaminophen-Propoxyphene
11689, 95178	Apap; Butalbital; Caffeine; Codeine
44, 3078, 7467, 93351	Apap; Caffeine; Dihydrocodeine
12555, 12565, 12570, 15983, 40020	Asa; Butalbital; Caffeine; Codeine
30340	Asa; Caffeine; Dihydrocodeine
4215, 8480, 10120, 25505, 25515, 25520, 28345, 41375	Asa; Caffeine; Propoxyphene
5018	Aspirin; Buffers; Codeine
10285	Aspirin; Caffeine; Dover’s Powder
105, 2803, 2820, 11230, 11235, 11240, 11245, 11250, 11255, 11260	Aspirin-Codeine
8397, 92181, 93027	Aspirin-Hydrocodone
1099, 2828, 22307, 22308, 23390, 23395, 58273, 93250	Aspirin-Oxycodone
30530	Aspirin-Pentazocine
8485, 8495	Aspirin-Propoxyphene
2943, 2955	Atropine-Meperidine
19655	Atropine-Morphine
3245, 21865	Belladonna-Opium
9516	Bupivacaine-Hydromorphone
3276	Buprenorphine-Naloxone
15650, 89034	Droperidol-Fentanyl
9737, 9751, 98043	Hydrocodone-Ibuprofen
5040	Ibuprofen-Oxycodone
1098, 4534, 8093, 18755, 96012	Meperidine-Promethazine
4538	Naloxone-Pentazocine

Statistical Package for Social Scientists (SPSS, IBM, Chicago, IL, version 21.0) was used to complete all statistical analyses and alpha was set at p < 0.05. Bivariate contingency table analysis was conducted to establish the relationships between each of the covariates and the dependent variable. Bivariate analysis tests for a statistically significant relationship between an outcome or dependent variable and a predictor or independent variable. Bivariate analysis is not a stratified analysis. SPSS allows an unadjusted odds ratio to be computed from the contingency table analysis as long as the contingency table is a 2 × 2 table. If not, then a chi square may be computed as the test statistic for differences between percentages (e.g., 3-group age ranges and opioid prescription). Logistic regression analysis, to produce adjusted measures, was performed using SPSS (version 21.0) complex samples. The complex samples algorithm was used to account for the stratified, clustered, and weighted variables in the 2010 NAMCS survey data. This was essential since NAMCS data are collected using a survey design where a nationally representative sample of physicians and practice sites are examined and sample weights applied to obtain national population-based statistics. Detailed documentation of the NAMCS instrument, methodology, and data files that served as the basis for this study is available elsewhere [25]. The multivariate logistic regression model was performed using opioid prescription as the dependent variable, and adjusted odds ratios were produced to test significance and establish effects sizes. US adults with a diagnosis of NMCP was the population examined. For analysis the sample of 2,745 was weighted to represent 9,325,603 US adults with a diagnosis of NMCP.

The Institutional Review Boards (IRBs) at all of the researchers’ institutions recognize that the analysis of de-identified, publicly available data does not constitute human subjects research as defined in federal regulations and as such does not require IRB review. Hence, human subjects’ approval was not necessary since this was a de-identified data only study.

## Results

An estimated 9,325,603 US adults with a diagnosis of NMCP were seen in primary care clinics in 2010 in the US. Table 2 displays data describing the study population --- US adults with NMCP. The data are displayed by the independent covariates and the dependent variable (opioid prescription) and are those used to perform the complex samples logistic regression analysis. Missing data were removed from the analysis as displayed in Table 2.

**Table 2:** **Description of the study population** (**adults with NMCP**) **for SPSS complex samples logistic regression analysis**

**Variables and Factors**		Weighted N*	**Weighted %**
Patient sex	Female	6868340	73.7
	Male	2457263	26.3
Patient age	18-39	565172	6.1
	40-64	4070522	43.6
	> = 65	4689909	50.3
Race/Ethnicity	Caucasian	7406401	79.4
	Non-Caucasian	1919202	20.6
Education percent of university graduates in patient zip code	<20%	5001646	53.6
	> = 20%	4323957	46.4
Poverty percent in patient zip code	<10%	4988235	53.5
	> = 10%	4337368	46.5
Health Insurance status	Have Health Insurance	8805283	94.4
	Do Not Have Health Insurance	520320	5.6
Primary HCP visit	Yes	6195674	66.4
	No	3129929	33.6
Patient now has arthritis	No	5087784	54.6
	Yes	4237819	45.4
Patient now has depression	No	7726881	82.9
	Yes	1598722	17.1
Geographic locale of patient	Rural	1966383	21.1
	Non-Rural	7359220	78.9
Opioid prescription**	Other Medications	5928705	63.6
	Opioids	3396898	36.4

*This weighted n is derived from a sample size of 2745, of which 2272 (82.8%) were non-rural residents and 473 (17.2%) rural residents.

**Study Dependent Variable.

NAMCS 2010 Data (weighted n =9,325,603).

The majority of the population 94.4% had health insurance and 66.4% were reported as having seen their primary HCP. Over seventy percent (73.7%) of the study population were women and 79.4% were Caucasian. In terms of the dependent variable 36.4% of the adult population with NMCP had an opioid prescription.

Bivariate analysis performed indicated that all of the study’s independent variables or covariates were significantly associated with the dependent variable (Table 3). Most importantly this bivariate analysis revealed that rural adults with NMCP had higher odds (OR =1.515, 95% CI =1.513 – 1.518) than similar non-rural adults of having a prescription for opioids.

**Table 3:** **Bivariate analysis of US adults with a diagnosis of chronic pain and an opioid prescription as dependent variable by covariates**

**Variable**	**Factor**	**Unadjusted odds ratio (95% CI)**	
Patient sex (vs. Male)	Female	1.107 (1.104, 1.109)	
Patient Race/Ethnicity (vs. Non-Caucasian)	Caucasian	.643 (.642, .644)	
Education percent university graduate in patient zip code (vs. > = 20% )	<20%	1.010 (1.008, 1.012)	
Poverty percent in patient zip code (vs. > = 10%)	<10%	1.036 (1.034, 1.037)	
Health Insurance status (vs. Do Not Have Health Insurance)	Have Health Insurance	1.010 (1.006, 1.014)	
Primary HCP visit (vs. No)	Yes	1.192 (1.190, 1.194)	
Patient now has arthritis (vs. Yes)	No	.885 (.883, .886)	
Patient now has depression (vs. Yes)	No	1.299 (1.295, 1.302)	
Geographic local (vs. Non-Rural)	Rural	1.515 (1.513, 1.518)	
**Variable**	**Factors**	%	**Significance**
Patient age range	18-39	6.1	< .001
	40-64	43.6	
	> = 65	50.3	

2010 NAMCS (weighted n =9,325,603).

Complex samples logistic regression was performed and the results are displayed in Table 4. Based on the bivariate analysis all of the study covariates were entered into the complex samples logistic regression model. Analysis yielded that two covariates were significantly associated with the dependent variable---rural residency (AOR =2.935, 95% CI 1.416-6.083) and non-Caucasian race/ethnicity (AOR =2.459, 95% CI 1.194-5.066).

**Table 4:** **SPSS complex samples logistic regression analysis of US adults with NMCP** (**study dependent variable** = **opioid prescription**)

**Variables**	**Factors**	**Adjusted odds ratio** **(95%** **CI)**
Patient sex	Female	1.310 (.631, 2.720)
	Male	--*
Patient age	18-39	1.094 (.297, 4.027)
	40-64	1.949 (.977, 3.887)
	> = 65	--*
Race/Ethnicity	Caucasian	--*
	Non-Caucasian	2.459 (1.194, 5.066)
Education percent of university graduates in patient zip code	<20%	--*
	> = 20%	1.031 (.489, 2.175)
Poverty percent in patient zip code	<10%	1.713 (.876, 3.351)
	> = 10%	--*
Primary HCP visit	Yes	1.162 (.515, 2.621)
	No	--*
Health Insurance status	Have Health Insurance	1.371 (.584, 3.221)
	Do Not Have Health Insurance	--*
Patient now has arthritis	Yes	1.309 (.514, 3.333)
	No	--*
Patient now has depression	Yes	.518 (.246, 1.089)
	No	--*
Geographic locale	Rural	2.935 (1.416, 6.083)
	Non-Rural	--*

*Reference category.

NAMCS 2010 data (weighted n =9,325,603).

## Discussion

Recently there has been much concern expressed about the over-prescribing of opioids [7,28–30]. This concern arises from the fact that opioids are potentially addictive which can lead to misuse. Opioids have been cited as contributing to unnecessary morbidity and mortality, and in the long-run contribute to potentially unnecessary medical costs [4,28]. We were interested in examining differences in rural and non-rural opioid prescribing patterns for NMCP management, hypothesizing that disparities exist in opioid prescribing patterns for rural populations in the US. Our analyses supported our hypothesis that rural residency is an independent risk factor for a greater probability of patients with NMCP being prescribed an opioid.

This research yielded important findings. First, rural residents had higher odds of having an opioid prescription than similar non-rural adults. Rural residency was the strongest predictor for having an opioid prescription and a diagnosis for NMCP. Second, being non-Caucasian was a strong predictor of having an opioid prescription and a diagnosis for NMCP.

Our results clearly indicate that rurality is an important factor in opioid prescribing patterns that cannot be ignored and bears further investigation. This is in stark difference to the finding of an earlier paper [24] that found no relationship between opioid prescription and geographic locale. At first glance one might conclude that this finding indicates an opioid prescribing disparity, but that may be too simple an explanation. All differences are not disparities. Disparities arise when the differences are avoidable as well as unjust [3,18]. We cannot say with any certainty that these are the characteristics of the differences revealed in prescribing patterns from our analyses. It may be that there are treatment option limitations in rural areas of the US [31]. Ultimately, NMCP is complex and often requires a multifactorial approach for optimal management [32]. For instance, physical therapy, occupational therapy, massage therapy, acupuncture, integrated specialty pain management services, or behavioral modification may be useful approaches for the management of patients with NMCP [33]. These modalities as treatment options may be less available to patients in the rural US [31].

Our findings also identified that non-Caucasian race/ethnicity (African American, Hispanic, Asian, Native American, and multiracial) was an independent risk factor for having NMCP and being prescribed an opioid. Opioid prescribing patterns for non-Caucasian adults is complicated and the differences between our findings and those from a number of other studies are hard to reconcile or explain. Findings from multiple studies have yielded that African American or Black patients were less likely than Caucasian ones to be prescribed opioids for pain [12,15,16,19,34–37]. Other research has revealed that in the US there is little difference in the estimated prevalence of pain across population groups [38]. However, racial/ethnic minorities have often had inadequate pain management despite being more likely to report experiencing severe pain and/or pain that interfered with daily activities [3,38]. One explanation for the variance of our findings from other studies might be the examination of data from a different source. The NAMCS data analyzed in this study is derived from patient health records rather than patient self-report surveys. Patient health record data are based on documented visits to health care providers, while patient self-report data are not. We do not want to make a claim of the veracity of one source of data over the other, only that the analysis of each may yield different findings.

### Study limitations and strengths

This study does have some limitations, most of which are attributable to how the survey data were collected. First, ICD-9 codes were used to identify patients with NMCP and to limit the population included in the study. Second, opioid prescriptions were then assumed to be linked to the NMCP patient population. Since there is no link in the questionnaire form between prescribed medications (in our case opioids) and diagnosis (ICD-9 codes), we were unable to determine with surety for what diagnosis opioids were being prescribed for. However, this limitation is also present in a previously published study [4], setting precedence for using this methodology. Studies that can determine causality amongst these variables are warranted. Third, the variables of race/ethnicity, education, and household income were derived variables.

Nevertheless, this study has a number of strengths. Since we used national patient record population-level survey data, we had a large data set that was weighted to ensure that our findings could be more easily and accurately generalized to the US population. Another strength is the magnitude of the effect sizes for rural and non-rural opioid prescribing patterns as well as those detected for race/ethnicity. The effect sizes, derived from the logistic regression odds ratios, ranged from nearly two to three times greater than the reference categories. Much has been studied in reference to age and race/ethnicity, but these findings lend vigorous support to the conceptualization of rurality as a social determinant of health.

## Conclusions

This study fills an important epidemiological knowledge gap regarding opioid prescribing patterns for rural adults with NMCP. Further research on the growing concern about the over-prescribing of opioids in the US should now include rurality as a variable in data generation and analysis in addition to the variable of race/ethnicity that are commonly included. In order to provide the best level of care to all patients regardless of geographic location or race/ethnicity, another level of analysis should capture data on opioid dosing and health care provider perceptions of patients. Future research should also attempt to document the ecological, sociological and political factors impacting opioid prescribing and care in rural communities. Prescribers and health care policy makers need to critically evaluate the implications of our findings and their relationship to patient needs, best practices in a rural setting, and the overall consequences of increased opioid prescribing on rural communities.

## Abbreviations

AOR: Adjusted odds ratios
CDC: Centers for disease control and prevention
HCP: Health care provider
IRB: Institutional Review Board
MSA: Metropolitan Statistical Area
NAMCS: National Ambulatory Medical Care Survey
NCHS: National Center for Health Statistics
NMCP: Non-Malignant Chronic Pain
OR: Odds ratios
SPSS: Statistical Package for Social Scientists
